# Small RNA sequencing reveals miR-642a-3p as a novel adipocyte-specific microRNA and miR-30 as a key regulator of human adipogenesis

**DOI:** 10.1186/gb-2011-12-7-r64

**Published:** 2011-07-18

**Authors:** Laure-Emmanuelle Zaragosi, Brigitte Wdziekonski, Kevin Le Brigand, Phi Villageois, Bernard Mari, Rainer Waldmann, Christian Dani, Pascal Barbry

**Affiliations:** 1 Centre National de la Recherche Scientifique, Institut de Pharmacologie Moléculaire et Cellulaire, UMR-6097, 660 route des lucioles, Valbonne Sophia-Antipolis, 06560, France; 2 University of Nice Sophia-Antipolis, 28 avenue Valrose, Nice Cedex 2, 06103, France; 3 Centre National de la Recherche Scientifique, Institut de Biologie du Développement et Cancer, UMR6543, 28 avenue de Valombrose, Nice cedex 2, 06107, France

## Abstract

**Background:**

In severe obesity, as well as in normal development, the growth of adipose tissue is the result of an increase in adipocyte size and numbers, which is underlain by the stimulation of adipogenic differentiation of precursor cells. A better knowledge of the pathways that regulate adipogenesis is therefore essential for an improved understanding of adipose tissue expansion. As microRNAs (miRNAs) have a critical role in many differentiation processes, our study aimed to identify the role of miRNA-mediated gene silencing in the regulation of adipogenic differentiation.

**Results:**

We used deep sequencing to identify small RNAs that are differentially expressed during adipogenesis of adipose tissue-derived stem cells. This approach revealed the un-annotated miR-642a-3p as a highly adipocyte-specific miRNA. We then focused our study on the miR-30 family, which was also up-regulated during adipogenic differentiation and for which the role in adipogenesis had not yet been elucidated. Inhibition of the miR-30 family blocked adipogenesis, whilst over-expression of miR-30a and miR-30d stimulated this process. We additionally showed that both miR-30a and miR-30d target the transcription factor RUNX2, and stimulate adipogenesis via the modulation of this major regulator of osteogenesis.

**Conclusions:**

Overall, our data suggest that the miR-30 family plays a central role in adipocyte development. Moreover, as adipose tissue-derived stem cells can differentiate into either adipocytes or osteoblasts, the down-regulation of the osteogenesis regulator RUNX2 represents a plausible mechanism by which miR-30 miRNAs may contribute to adipogenic differentiation of adipose tissue-derived stem cells.

## Background

Obesity, by itself or associated with ancillary disorders such as diabetes and cardiovascular pathologies, represents a major public health issue in developed countries. In severe obesity, as well as in normal development, the growth of adipose tissue is the result of adipocyte hypertrophy and hyperplasia. It is now well established that a pool of multipotent progenitor cells persists in adipose tissue throughout life and is able to differentiate to give rise to adipocytes [1-3]. Certain key events controlling the terminal differentiation of progenitors into adipocytes have been identified. Transcription factors such as CCAAT/enhancer-binding proteins (C/EBPs) and peroxisome proliferator-activated receptors (PPARs) are known to play a critical role in this process [4]. However, the molecular mechanisms controlling the early steps of adipocyte progenitor commitment towards adipocyte differentiation remain poorly understood. Several lines of evidence suggest that osteoblasts and adipocytes share the same precursor cell type. Mesenchymal stem cells isolated from different tissues can differentiate into both lineages at a clonal level [2,5,6]. A reciprocal and inverse relationship exists between adipogenesis and osteogenesis [7-9]. Pathophysiological conditions such as ageing or osteoporosis, for instance, involve a concomitant decrease in trabecular bone volume and an increase in bone-marrow adipocyte numbers [10]. Moreover, molecular mechanisms that activate differentiation towards one lineage often inhibit differentiation towards the opposite fate. Several signaling pathways, including the bone morphogenetic protein, Wnt, Hedgehog and insulin-like growth factor pathways, as well as transcription factors such as PPARγ and RUNX2 (runt-related transcription factor 2), have already been shown to modulate the balance between adipogenesis and osteogenesis (reviewed in [11]).

MicroRNAs (miRNAs) are a subclass of regulatory, non-coding RNAs that regulate gene expression at a post-transcriptional level by affecting mRNA translation and stability [12]. Up to 30% of human genes could potentially be regulated by miRNAs [13]. The ability of a miRNA to interact with many targets, together with the possibility for several miRNAs to share the same target, represent powerful regulatory mechanisms that tremendously increase the complexity of biological networks. Over the past few years, miRNAs have been shown to regulate many cellular processes, including adipogenesis and osteogenesis. miR-103, miR-143, miR-17~92, miR-21, and miR-204/211 have been reported to promote adipogenesis [14-18], while the miR-27 family inhibits this process [19]. Similarly, osteogenesis is regulated positively by miR-29b, and negatively by miR-133, miR-135 and miR-125b [20].

Our present work aims to clarify the role of miRNAs in the regulation of adipogenesis. We have characterized small RNAs that are modulated by adipogenic differentiation in human adipose tissue-derived stem (hMADS) cells by a deep-sequencing approach. Among the RNA species we sequenced, miRNAs were the most abundant class of annotated small RNAs. However, we also found significant variations in expression levels of non-annotated small RNAs during adipogenic differentiation. A current bioinformatics challenge in small RNA research is the prediction of RNA targets and how their regulation is integrated into already existing biological networks. We performed such a study in the specific context of the miR-30 family, in order to evaluate the capacities of these miRNAs to regulate adipogenesis. Our investigations focused on the transcription factor RUNX2, a major regulator of osteogenesis, which we established as a *bona fide *target of miR-30a and miR-30d.

## Results

### Global analysis of miRNAs by high-throughput sequencing during adipogenesis of hMADS cells

To identify small RNAs that are differentially expressed during human adipogenesis, hMADS cells were differentiated into adipocytes *in vitro*. RNA was extracted from confluent undifferentiated (day 0) cells and from cells that were differentiated for 3 or 8 days. Differentiation efficiency was checked by expression profiling of specific genes, such as those encoding adiponectin and PPARγ (Additional file 1).

**Figure 1:**
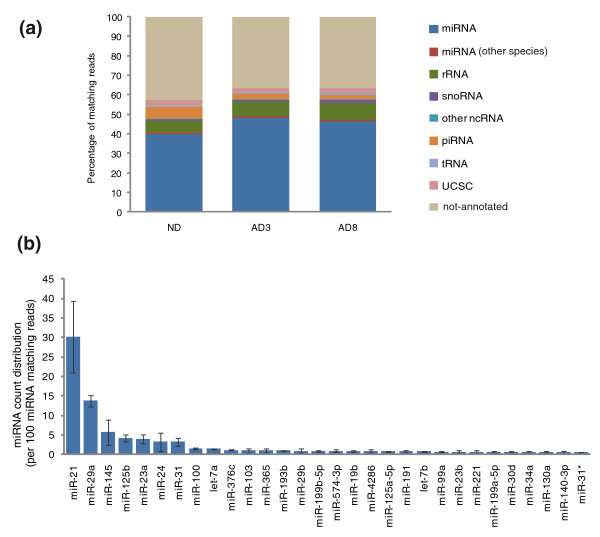
**Distribution of deep-sequenced small RNAs across non-coding RNA categories**. **(a) **Reads were matched versus the hg19 genome build and then distributed in an exclusive manner to human miRNAs, as well as miRNAs of species other than human (mirBase 16), to UCSC annotated sequences (UCSC Refflat file) and finally to non-coding RNA classes (fRNAdb, database of ncRNA.org): piwi-interacting RNA (piRNA), tRNA, rRNA, small nucleolar RNA (snoRNA) and other non-coding RNA (ncRNA). Reads that did not match any of those non-coding RNA classes were labeled as 'non-annotated'. Data are the average of read sequencing frequency (percentage) for each experimental condition. ND, undifferentiated cells; AD3, adipogenesis day 3; AD8, adipogenesis day 8. **(b) **Relative abundance of reads corresponding to the 30 most expressed miRNAs in undifferentiated hMADS cells. Read counts are normalized to 10^6 ^total miRNA reads per sample. Data are the average of sequencing of samples from two independent experiments, each with two technical replicates with opposite sequencing directions (error bars represent ± standard error).

Small RNA libraries were sequenced on an Applied Biosystems SOLiD sequencer. As shown in Figure 1a, 40 to 45% of the reads that were mapped to the human genome (release hg19) accounted for miRNAs annotated in mirBase (release 16). Other small RNA species, such as piwi-interacting RNAs (piRNAs) and small nucleolar RNAs (snoRNAs), were also identified but with a lower abundance. Interestingly, 36.5 to 42.6% of mapped reads corresponded to non-annotated small RNAs. The distribution of the different miRNAs was highly heterogeneous: just a few miRNAs represented high fractions of the reads. For instance, in undifferentiated cells, among the 145 mature miRNAs that each represented > 0.03% of the reads, 131 had a relative abundance that was below 1% while miR-21 and miR-29a were highly abundant and accounted for 30.2% and 13.8% of miRNA reads, respectively (Figure 1b). The complete set of detected mature miRNAs is shown in Additional file 2.

**Figure 2:**
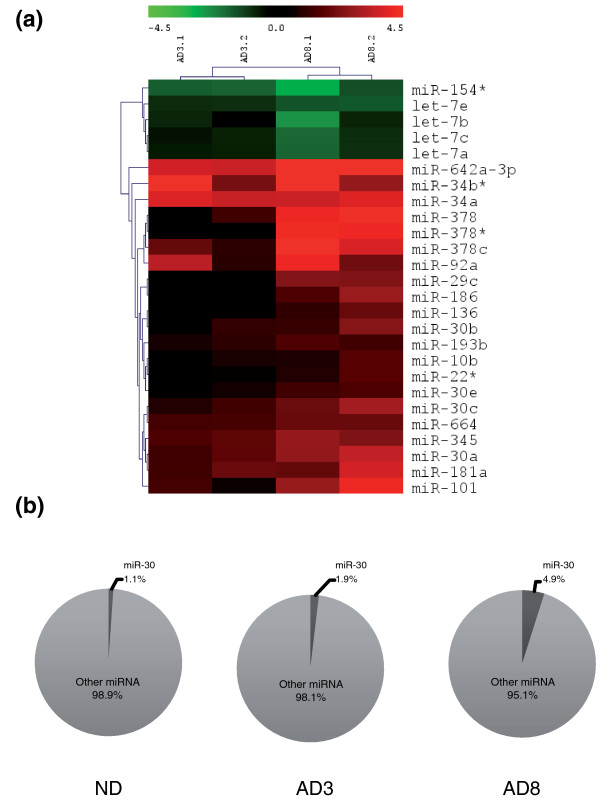
**miRNA expression data in differentiated versus undifferentiated human adipose tissue-derived stem cells**. **(a) **Heatmap of the fold-change (log2 transformed) of miRNA expression in differentiated versus undifferentiated hMADS cells. The top 26 regulated miRNAs are represented (*P*-value < 0.05). Two independent experiments are displayed. **(b) **Relative abundance of the miR-30 family over total detected miRNAs in undifferentiated and adipocyte-differentiated hMADS cells. Data are the average of sequencing of samples from two independent experiments, each with two technical replicates with opposite sequencing directions. ND, undifferentiated cells; AD3.1, adipogenesis day 3, replicate 1; AD3.2, adipogenesis day 3, replicate 2; AD8.1, adipogenesis day 8, replicate 1; AD8.2, adipogenesis day 8, replicate 2.

The relative abundance of each miRNA was then compared between differentiated (adipogenesis at day 3 and day 8) and undifferentiated (confluency) conditions. For statistical analyses, only miRNAs with a minimum relative abundance of 0.03% in at least one of the experimental condition were considered. A significant differential expression was observed for 26 miRNAs, based on a *P*-value below 0.05. This defined our top 26 regulated miRNAs, the expression pattern of which is depicted in Figure 2a and Table 1. Twenty-one miRNAs from the top 26 were up-regulated during differentiation, while five miRNAs were down-regulated. Thus, differentiation seems to be characterized by a predominant increase in miRNA expression.

**Table 1:** Top 26 regulated miRNAs during adipogenesis

	**log**_**2**_**(AD3/ND)**	**log**_**2**_**(AD8/ND)**	*P*-value AD8/ND	Maximal read number across all samples (per million miRNA reads)
hsa-miR-642a-3p^a^	3.64 ± 0.04	7.32 ± 0.08	4.67E-07	349
hsa-miR-378	0.72 ± 0.42	4.32 ± 0.11	2.22E-06	8,714
hsa-miR-378*	0.28 ± 0.02	4.24 ± 0.05	9.42E-05	998
hsa-miR-345	1.68 ± 0.10	2.53 ± 0.13	3.76E-04	1,605
hsa-miR-378c	1.49 ± 0.32	4.51 ± 0.49	0.001	698
hsa-miR-193b	0.89 ± 0.10	1.46 ± 0.08	0.005	31,446
hsa-miR-29c	0.43 ± 0.01	2.41 ± 0.2	0.007	26,009
hsa-miR-34b*	4.10 ± 1.34	4.51 ± 1.28	0.007	1,009
hsa-let-7e	-0.85 ± 0.02	-1.48 ± 0.03	0.008	1,338
hsa-miR-30c	1.09 ± 0.18	2.49 ± 0.33	0.009	7,848
hsa-miR-664	1.41 ± 0.01	1.98 ± 0.01	0.012	739
hsa-miR-10b	0.56 ± 0.15	1.25 ± 0.31	0.019	3,258
hsa-miR-34a	3.70 ± 0.12	3.73 ± 0.11	0.022	32,529
hsa-miR-30a	1.54 ± 0.18	3.05 ± 0.27	0.022	49,240
hsa-miR-186	0.36 ± 0.03	2.15 ± 0.46	0.023	845
hsa-miR-136	-0.25 ± 0.02	1.53 ± 0.33	0.027	3,083
hsa-miR-30b	0.53 ± 0.42	1.82 ± 0.46	0.027	3,185
hsa-let-7b	-0.47 ± 0.18	-1.57 ± 0.60	0.032	13,065
hsa-miR-22*	0.41 ± 0.06	1.27 ± 0.31	0.034	1,281
hsa-miR-30e	0.52 ± 0.13	1.41 ± 0.08	0.037	2,251
hsa-miR-181a	1.66 ± 0.24	2.82 ± 0.64	0.041	5,101
hsa-miR-154*	-1.64 ± 0.03	-2.09 ± 0.52	0.043	367
hsa-miR-92a	2.14 ± 0.83	3.14 ± 0.73	0.043	2,885
hsa-let-7c	-0.58 ± 0.07	-1.3 ± 0.33	0.044	1,718
hsa-let-7a	-0.62 ± 0.07	-1.29 ± 0.29	0.045	19,329

miRNAs were sorted according to decreasing AD8-ND *P*-value (*P *< 0.05). AD3, adipogenesis day 3; AD8, adipogenesis day 8; ND, not differentiated. ^a^Mature miRNA is not annotated in mirBase (pre-miR is annotated).

The expression patterns of miRNAs that were previously reported in adipocytes or their precursors are in agreement with published data, as summarized in Additional file 3. However, the adipogenesis-dependent regulation of many of the differentially expressed miRNAs we identified has never been described before; these include miR-642a-3p, miR-345, miR-193b, miR-29c, miR-664, miR-10b, miR-136, miR-22*, miR-181a, miR-154*, let-7a, let-7b and let-7c.

### Up-regulation of miR-642a-3p, miR-378/378* and miR-30 miRNAs suggests their contribution to adipogenesis

The expression profile of the miRNAs that were strongly up-regulated during adipogenesis (miR-642a-3p, miR-378, miR-30a, miR-30b, miR-30c, miR-30d, miR-30e, and miR-193b) was validated by quantitative PCR (qPCR; Additional file 4). Although some of the fold changes obtained by this technique were not strictly equal to those obtained by deep sequencing, this approach confirmed qualitatively the stimulation of the expression for all of these miRNAs.

**Figure 3:**
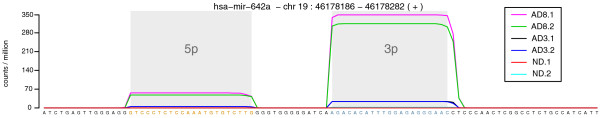
**Abundance of each base along the miR-642a pre-miR**. Each experimental condition is pictured using the color code in the insert. Light grey shading highlights miR-642-5p (bases in orange) and miR-642-3p (bases in blue). Represented samples were sequenced in the 3' to 5' direction.

miR-642a-3p, with a 7.32-fold induction during adipogenic differentiation, was the most highly and significantly (*P*-value = 4.67.10^-7^) regulated miRNA in our dataset (Table 1 and Figure 2a). Of note, miR-642a-3p is not annotated in mirBase 16; only miR-642a-5p has been reported before. In our dataset, both miR-642a-5p and -3p were induced during differentiation, but miR-642a-3p had a higher relative abundance than miR-642a-5p (Figure 3). For identification of differentially expressed miRNAs, only miR-642a-3p reached significance since the cloning frequency of miR-642a-5p was under the threshold of 0.03% that we defined. Interestingly, both miR-642a-3p and miR-642a-5p were undetectable in undifferentiated hMADS cells, suggesting a high specificity for adipocytes. miR-642a is positioned on chromosome 19, in intron 7 of the *GIPR *(glucose-dependent insulinotropic polypeptide receptor) gene (Additional file 5). *GIPR *mRNA and protein were found to be up-regulated during adipocyte differentiation [21]. This is consistent with an up-regulation of miR-642a, assuming that miR-642a and *GIPR *share the same promoter. The GIPR ligand, GIP, was shown to promote fatty acid synthesis in adipocytes [22] and to favor obesity *in vivo *[23]. Altogether, these data suggest that miR-642a might be linked to adipose tissue development.

Incidentally, miR-378 microRNAs, also highly regulated in our model, have a genomic location in intron 1 of *PPARGC1B *(Additional file 5) and miR-378 has already been described as positively regulated in adipogenesis (Additional file 3). In addition to miR-378, our data confirmed that the miR-30 family was up-regulated in adipogenesis (Table 1). Interestingly, the relative abundance of the miR-30 family varies from 1.1% in undifferentiated cells to 4.9% in adipocyte-differentiated cells (Figure 2b). In particular, miR-30a and miR-30d accounted for 3.7% of all sequenced miRNAs in adipocyte-differentiated hMADS cells. Even though none of the miR-30 family members are encoded within introns of pro-adipogenic sites, their increased abundance is likely to reflect a major role in differentiation.

### Gain and loss of function studies reveal that the miR-30 family favors adipogenesis

Given their up-regulation after induction of adipogenesis and their high abundance in adipocytes, we focused on the role of miR-30 family members in adipogenesis. We altered their expression by transfecting synthetic miR-30 miRNAs or the corresponding antagomirs. Inhibition of the miR-30 family was achieved with the transfection of a combination of three oligonucleotides that can target and inhibit activity of the whole miR-30 family. Over-expression was obtained with transfection of pre-miRNAs for miR-30a and miR-30d. In both cases, sub-confluent hMADS cells were transfected and then submitted to adipogenic differentiation three days later, once cells had reached confluency. Adipogenesis was scored after 10 days (miRNA knock-down) or 4 days (miRNA over-expression) in differentiating medium. At each analyzed time point, inactivation and over-expression were efficient, as shown by qPCR (Additional file 6).

**Figure 4:**
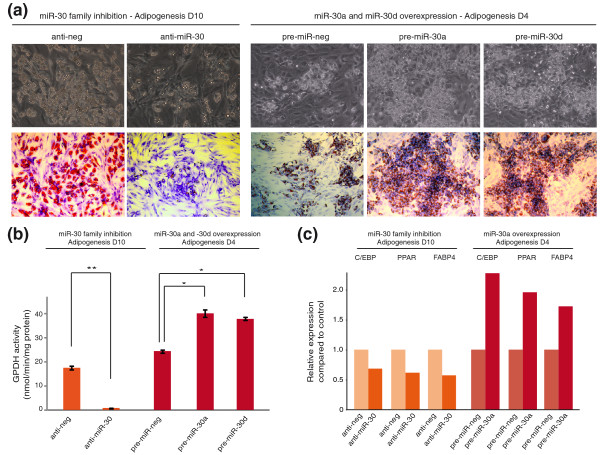
**The miR-30 family positively regulates hMADS cell adipocyte differentiation**. **(a) **Sub-confluent hMADS cells were transfected with one of anti-miR control (anti-neg), anti-miR 30, pre-miR control (pre-miR-neg), pre-miR-30a, or pre-miR-30d and were induced to undergo adipocyte differentiation 3 days later. Differentiation was assessed at the indicated time points (D4 or D10) by photomicrographic recording (top row) and Oil red O plus crystal violet counter-staining (lower row). **(b) **Assessment of adipogenesis by GPDH enzymatic activity. Results are means of three culture wells (24-well plates). Error bars represent mean ± standard error of the mean (*N *= 3). *P < 0.05. **(c) **Expression of adipogenesis-induced genes (*CEBPβ, PPARγ *and *FABP4*) by qPCR. The level of expression of each gene in control cells (anti-miR-neg or pre-miR-neg) was taken as 1.

Morphological observation and coloration of lipid droplets showed that inactivation of the miR-30 family impaired adipogenesis and that over-expression of miR-30a and miR-30d improved adipogenesis (Figure 4a). This was confirmed by the evaluation of the adipogenic-specific glycerol-3-phosphate deshydrogenase (GPDH) enzymatic activity. Inactivation of the miR-30 family drastically reduced GPDH activity at day 10 (fold reduction of 23.9). Interestingly, over-expression of miR-30a and miR-30d was sufficient to enhance this activity at day 4 (fold induction of 1.6; Figure 4b). Finally, we checked for the expression of the adipogenic-induced transcripts C/EBPβ, PPARγ and fatty acid binding protein (FABP) 4. These genes showed consistent profiles after inactivation of the miR-30 family and over-expression of miR-30a (Figure 4c).

### miR-30 miRNAs stimulate adipogenesis via inhibition of the osteogenesis transcription factor RUNX2

To identify molecular mechanisms that would regulate the effects of miR-30 miRNAs on adipogenesis, bioinformatics prediction of their targets was performed with TargetScan. It revealed that RUNX2 bears several conserved binding sites for these miRNAs (Additional file 7). RUNX2, also known as CBFA1, is a key regulator of osteogenesis and its expression is detected at the undifferentiated state. It increases during osteogenesis and decreases during adipogenesis [24] (Additional file 1).

**Figure 5:**
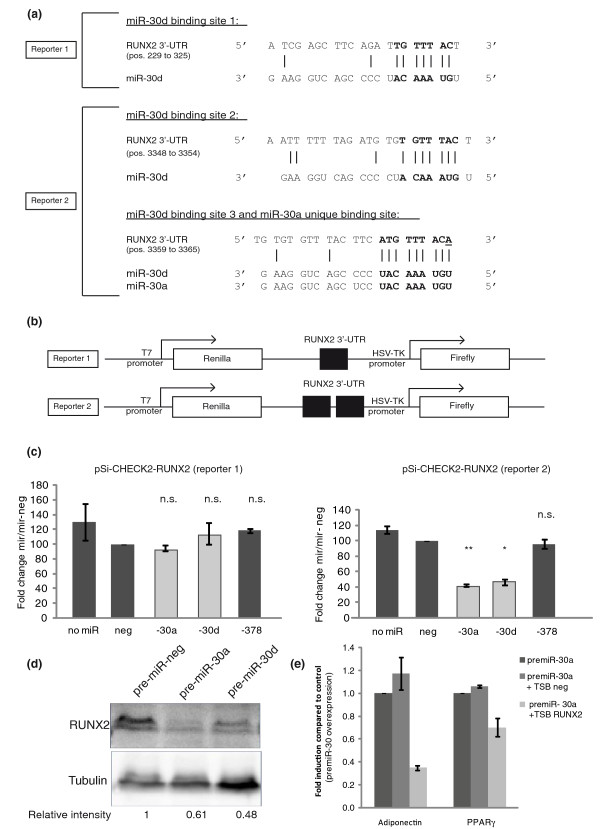
***RUNX2 *mRNA is a primary target for miR-30a and miR-30d**. **(a) **Predicted interaction between miR-30a and miR-30d and their putative binding sites in the 3' UTR of *RUNX2*. The representation is limited to the region around the miR-30a and miR-30d complementary sites. In bold is the 'seed' region with a conserved anchoring adenosine that is complementary to the first nucleotide of miR-30a and miR-30d (underlined). **(b) **Schematic representation of the construct used in the luciferase assay: a 300-bp (reporter 1) and 319-bp (reporter 2) region of the 3' UTR of human *RUNX2 *containing the putative miR-30a and/or miR-30d target sites (black boxes) were cloned into the pSi-CHECK™-2 vector. **(c) **Normalized luciferase activity 48 hours after co-transfection of human pre-miR-30a, pre-miR-30d, pre-miR-378 or pre-miR-control (neg) together with pSi-CHECK™-2 constructs in HEK 293 cells. Data were obtained from four independent experiments (error bars represent average ± standard error); n.s., not significant compared to pre-miR-control; *significant compared to pre-miR-control (*P *< 0.05); **significant compared to pre-miR-control (*P *< 0.01). **(d) **Undifferentiated hMADS cells were transfected with either pre-miR control (pre-miR-neg) or pre-miR-30a or pre-miR-30d. Four days later, cell lysates were prepared and expression of RUNX2 was investigated by western blotting. Tubulin was used as a loading control. The integrated density of each band was quantified with Image J. Densities obtained for RUNX2 signals were divided by the corresponding tubulin densities. Numbers below the blot are the density fold changes compared to the control condition. **(e) **Undifferentiated hMADS cells were transfected with control target site blocker (TSB-neg) or with RUNX2 target site blocker (TSB-RUNX2) as well as with pre-miR-30a. Adipogenic differentiation was evaluated by analyzing adiponectin and *PPARγ *expression by qPCR. The level of expression of each gene in the pre-miR-30a condition was taken as 1.

In order to test whether RUNX2 is targeted by the miR-30 family, we cloned two regions of its 3' UTR that contain the predicted miR-30 binding sites into the pSi-CHECK™-2 vector, downstream of the Renilla translational stop codon (Figure 5a,b). The first region covers positions 32 to 332 of the RUNX2 3' UTR and contains a poorly vertebrate-conserved putative miR-30 binding site (positions 229 to 235 of the RUNX2 3' UTR). The second region covers positions 3,102 to 3,421 of the RUNX2 3' UTR and encompasses two vertebrate-conserved putative binding sites (positions 3,348 to 3,354 and positions 3,359 to 3,365 of the RUNX2 3' UTR).

HEK-293T cells were co-transfected with either construct together with the following synthetic pre-miRNAs: negative control, miR-30a, miR-30d or miR-378 (as RUNX2 does not bear any putative binding site for this miRNA, miR-378 was used here as an additional control). When cells were transfected with pSi-CHECK™-2 bearing the first putative binding site, none of the tested miRNAs had any effect on luciferase activity. In contrast, with pSi-CHECK™-2 bearing the last two binding sites, miR-30a and miR-30d triggered a more than two-fold decrease in luciferase activity compared to the control miRNA (Figure 5c). As expected, miR-378 had no effect on luciferase activity. Importantly, this effect was confirmed at the protein level for endogenous RUNX2. Transfection of sub-confluent hMADS cells with pre-miR-30a or pre-miR-30d induced a 0.61-fold or 0.48-fold decrease in RUNX2 protein levels, respectively (Figure 5d). Thus, these results demonstrate that RUNX2 is a *bona fide *target of miR-30a and miR-30d.

Finally, we sought to establish a direct link between miR-30 effects on adipogenesis and RUNX2 targeting. We used the target site blocker (TSB) strategy to mask miR-30 binding sites 2 and 3 in the *RUNX2 *3' UTR. Transfection with RUNX2 miR-30-specific TSB, but not a control TSB, significantly decreased miR-30a stimulation of adipogenesis (Figure 5E). In conclusion, RUNX2 targeting is, at least in part, responsible for miR-30 positive effects on adipocyte differentiation.

## Discussion

Adipocyte differentiation is a complex process combining several levels of regulation. Signaling pathways, such as cAMP and insulin signaling pathways, as well as key transcription factors, such as PPARγ, C/EBPβ and Krüppel-like transcription factors (KLFs), have been extensively studied [4,25].

Our results suggest a direct role of miRNA-mediated post-transcriptional regulation in adipogenesis. In particular, we show that the miR-30 family is a positive, key regulator of adipocyte differentiation in a human adipose tissue-derived stem cell model. The up-regulation of miR-30 expression is triggered at early stages of adipocyte differentiation (day 3) and increases until terminal differentiation. Of note, all miR-30 miRNAs do not belong to the same genomic cluster (Additional file 8). In particular, miR-30a and miR-30d are encoded by genes located on distinct chromosomes, suggesting coordinated regulation of distinct genomic regions. Factors that are responsible for this coordinated regulation have not yet been elucidated.

In order to dissect the molecular mechanisms involved in the effects of miR-30 on adipogenesis, we searched for predicted target genes. We focused on *RUNX2*, which is a well-established regulator of osteogenesis. Indeed, an inverse relationship is known to regulate the balance between adipogenesis and osteogenesis. Thus, identifying miRNAs that are up-regulated during adipogenesis and that negatively regulate a key osteogenesis transcription factor is of major importance. In fact, the RUNX2 pathway has been reported as a potent inhibitor of the expression of the master gene for adipogenesis, *PPARγ *[26]. Thus, it is tempting to speculate that RUNX2 inhibition is required for adipocyte differentiation and that miR-30 miRNAs play a critical role in this process.

We show here for the first time that miR-30 miRNAs target RUNX2. Huang and co-workers [18] recently demonstrated that miR-204 and miR-211, which were up-regulated during adipogenesis of human bone marrow stem cells, also target RUNX2. However, we found that miR-204 and miR-211 were expressed at extremely low levels - for example, below our 0.03% threshold - while miR-30 represented 4.9% of the miRNA reads in adipocytes. This is probably not due to a deep sequencing cloning bias, as miR-204 detection was above average and better than that of miR-30 in a synthetic equimolar miRNA panel that we sequenced in similar conditions (data not shown). Thus, in our system, this very low abundance of miR-204 and miR-211 suggests that their impact on RUNX2 and differentiation is minor when compared with the highly expressed miR-30 family. Importantly, we also showed that miR-30 stimulation of adipogenesis was impaired by masking miR-30 binding sites in the 3' UTR of *RUNX2*, and preliminary data suggest that miR-30 inhibition might stimulate osteogenesis. Altogether, these data strongly support a direct and functional link between RUNX2 and miR-30, but does not exclude the contribution of additional miR-30 targets. In an attempt to identify the ones that were regulated at the RNA level, we performed a transcriptome analysis of hMADS cells that were transfected with pre-miR-30a or pre-miR-30d and then submitted to adipocyte differentiation for 4 days. Using miRonTop [27], we verified that predicted miR-30 targets were correctly enriched in these experiments. Statistical scores were highest for the miR-30 family (*P*-value = 5.32.10^-10^), showing its strong overall impact in these cells. In the list of predicted miR-30 targets, we noticed the presence of CBFB (core binding factor beta), a co-transcription factor that forms a heterodimer with RUNX proteins [28]. CBFB was down-regulated after over-expression of miR-30a and -miR30d, and slightly up-regulated in the antimiR-30 condition. Since CBFB was shown to be essential for functions of RUNX1 and RUNX2 [28], these additional data may explain the drastic effect of miR-30 on adipogenesis.

In addition to miR-30 miRNAs, we identified potent up-regulation of other miRNA families, such as miR-378 (35.7-fold), during adipogenic differentiation. A role of decreased miR-378 expression in osteogenesis in the osteoblastic cell line MC3T3-E1 has been suggested recently [29]. Indeed, miR-378 appears to target nephronectin, which is a positive regulator of osteoblastic differentiation. Very recently, Gerin and co-workers [30] identified miR-378/378* as positive regulators of lipogenesis.

Although expressed at lower levels than the highly abundant miR-30 family, two members of the miR-642 family were the most highly up-regulated miRNA in our adipogenesis model. The function of these miRNAs has not been reported before. Of interest, in a recent study identifying the association of miR-519b with human obesity, Martinelli and co-workers [31] also detected that miR-642a was up-regulated in 19 out of 20 fat depots of obese subjects. In our data, no reads corresponding to miR-642a were detected for undifferentiated cells, indicating highly adipogenic-restricted expression. Amongst both miR-642a isoforms, only miR-642a-3p was above the 0.03% threshold in our model. Yet, until recently (September 2010), only miR-642a-5p was present in mirBase release 15 (named miR-642 in release 15) and, thus, detectable on commercial microarrays. The current mirBase release (release 17) includes two miR-642 entries: miR-642a (miR-642a-5p), which was detected at one copy in a unique, high-throughput sequencing experiment; and miR-642b, which is backed by an unknown number of reads.

As shown in Additional file 8, miR-642b is, in fact, located on the opposite strand to miR-642a. The mature sequence annotated in mirBase for miR-642b is the 3p arm of the pre-miRNA. While we also detected miR-642b, this sequence was much less (14-fold) abundant than miR-642a-3p. miR-642a-3p and miR-642b sequences are, in fact, quite similar and only diverge by one base in their 3' end. This observation raises doubts about the *bona fide *existence of miR-642b. In our dataset, the few reads that were attributed to miR-642b could, in fact, correspond to miR-642a-3p reads bearing sequencing errors. To support this hypothesis, we counted the reads attributed to each miR-642 species within the raw read files. This approach requires conversion of each miRNA sequence into the corresponding color-space sequence, and a perfect match search for these sequences in the read files. This method confirmed that miR-642b was detected at very low levels compared with miR-642a-3p (Table S4 in Additional file 9). We also verified the quality of miR-642a-3p sequencing. Figure S6 in Additional file 9 shows that the positions allowing discrimination of miR-642-3p from miR-642b correspond to high quality values. These values suggest that the corresponding reads were correctly assigned to miR-642-3p.

More generally, this raises questions about the quality of some mirBase annotations. In particular, for miRNAs with highly tissue-specific expression, such as miR-642a, the low numbers of reads backing the mirBase entries might lead to incorrect annotations.

Even though our study focused on miRNAs, we also noted that 34.2% of reads that were mapped to the reference genome did not correspond to any annotated small RNA. Our small RNA cloning strategy only captures small RNAs that are, as miRNAs, 5'-phosphorylated and, thus, eliminates RNA degradation products generated by the major cellular ribonucleases, which generate fragments that are not 5'-phosphorylated.

Some of those un-annotated, small RNAs were significantly regulated during adipogenesis (not shown). Most of the regulated sequences are located within the introns of annotated genes. For instance, we identified an adipocyte-enriched, 21-bp sequence within the fourth intron of NCOA2 (nuclear receptor coactivator 2, or transcriptional intermediary factor 2 (TIF2); Additional file 10). It is noteworthy that NCOA2 is associated with obesity. In fact, TIF2^-/- ^mice are resistant to diet-induced obesity and TIF^-/- ^mouse embryonic fibroblasts store lipids with a much lower efficiency than TIF2^+/+ ^mouse embryonic fibroblasts [32].

We also found that 2.6 to 6.3% of small RNA reads mapped to tRNA sequences. Recently, Lee and co-workers [33] described a new class of tRNA-derived small RNAs, termed tRFs, that are not products of random degradation or biogenesis. In our data, we found abundant reads matching the 5' end of mature tRNA (Additional file 10). No function for this class of small RNA has yet been suggested.

## Conclusions

We identified several annotated, but also previously unknown, small RNAs that are regulated during adipogenesis, such as miR-642a-3p. Deep sequencing also allowed the relative abundance of each miRNA to be estimated, revealing miRNAs that reach relatively high expression levels and are, thus, potentially relevant in adipogenesis. Amongst the adipogenesis-induced miRNAs, miR-30 reached the highest levels during differentiation. We show that this miRNA family plays an important role in adipogenesis via the targeting of RUNX2, a major regulator of osteogenesis.

## Materials and methods

### Cell culture

hMADS cells were obtained from the stroma of human adipose tissue as described previously [34]. Briefly, we used the stroma-vascular fraction of white adipose tissue from young donors (1 month old to 7 years old). Adipose tissue was collected, with the informed consent of the parents, as surgical scraps from surgical specimens from various surgeries, as approved by the Centre Hospitalier Universitaire Nice Review Board. Approximately 200 mg of adipose tissue were dissociated with type A collagenase and the stroma-vascular fraction was separated from the adipocyte fraction by centrifugation. The crude stroma-vascular fraction was plated on uncoated culture dishes; 12 hours after plating, non-adherent cells were removed by a medium change and adherent cells (termed CA by Rodriguez *et al. *[34]) were maintained in the proliferation medium, which is composed of DMEM (low glucose) containing 10% fetal calf serum, 0.01 M HEPES, 100 U/ml penicillin and streptomycin. The hMADS cell populations included in this study were isolated from a 4-month-old (hMADS) male [34]. HEK 293 cells were purchased from the American Type Culture Collection (Manassas, VA, USA) and maintained in monolayer culture in DMEM supplemented with 10% fetal calf serum.

### *In vitro *hMADS cell differentiation

Adipocyte differentiation was induced on the day hMADS cells reached confluency. Adipogenic medium was composed of DMEM/Ham's F12 media supplemented with 10 μg/ml transferrin, 0.86 μM insulin, 0.2 nM triiodothyronine, 1 μM dexamethasone, 100 μM isobutyl-methylxanthine and 1 μM rosiglitazone. Three days later, the medium was changed (dexamethasone and isobutyl-methylxanthine were omitted).

### Evaluation of hMADS cell adipocyte differentiation

Neutral lipid accumulation was evaluated by Oil red O staining, as previously described [35]. GPDH activity was performed in triplicate wells, using the method described previously [36] (GPDH is an enzyme that is required for the formation of triglycerides). Expression of the adipogenesis-induced markers PPARγ2, FABP4, adiponectin and C/EBPβ was also evaluated by real-time qPCR.

### RNA extraction

hMADS cells were lysed by addition of TRIZOL reagent (Invitrogen, Life Technologies Corporation, Carlsbad, CA, USA) on the cell layers. Total RNAs containing the small RNA fraction were then purified on a RNeasy kit column (Qiagen, Valencia, CA, USA) according to the manufacturer's instructions. Purity and concentration of total RNA samples were first evaluated using a Nanodrop spectrophotometer (Thermo Scientific, Waltham, MA, USA). RNA samples were run in a RNA nano-chip into a 2100 Bioanalyzer System (Agilent Technologies, Santa Clara, CA, USA) to verify the integrity of the RNA samples.

### Gene expression analysis by real-time qPCR and DNA microarray

RNAs were retro-transcribed with the Mirscript RT kit (Qiagen). Quantitative PCR was performed using LightCycler^® ^480 SYBR Green I Master mix and Light Cycler 480 real-time PCR machine (Roche Applied Science, Indianapolis, IN, USA). Expression levels of transcripts were evaluated using the comparative CT method (2-deltaCT). Transcript levels of *POLR2A *and *RPL13 *were used for sample normalization. Results are log2-transformed fold changes of normalized 2-deltaCT. Data were obtained from three independent experiments and are represented as average ± standard error. Primer sequences are detailed in Additional file 11.

DNA microarrays experiments were performed on Agilent Sureprint G3 Human GE 8x60K microarrays according to the manufacturer's instructions. The experimental data and microarray design have been deposited in the NCBI Gene Expression Omnibus [37] under series GSE29207.

### Small RNA cloning and sequencing

Total RNA containing the small RNA fraction were isolated from hMADS cells as described above. The SOLiD™ Small RNA Expression Kit (Applied Biosystems, Life Technologies Corporation, Carlsbad, CA, USA) was used to build a library of double-stranded DNA molecules from the population of small RNAs present in the different samples, which were then read using the Applied Biosystems SOLiD™ System sequencing according to the manufacturer's instructions. Briefly, total RNAs containing the small RNA fraction were hybridized (at 65°C for 10 minutes, then at 16°C for 5 minutes) and ligated (at 16°C for 16 hours) to adapters that are provided by the Small RNA Expression Kit. Adaptor mix A (AdA) and adaptor mix B (AdB) were used to produce templates for sequencing small RNAs from the 5' ends and from the 3' ends, respectively. As described in the Small RNA Expression Kit, samples were then reverse transcribed (at 42°C for 30 minutes) to synthesize cDNA and treated with RNAse H (37°C for 30 minutes). Small RNA libraries were amplified by PCR (17 cycles) and size selected on 8% polyacrylamide gels. The 105- to 150-bp material (corresponding to 15- to 50-bp small RNAs) was excised from the gel and eluted in nuclease-free water (70°C for 3 hours). DNA concentrations of all samples were measured by qPCR.

Libraries were amplified by emulsion PCR and sequenced on SOLiD according to the manufacturer's instructions. Read length was 35 bp. The experimental data have been deposited in the NCBI Gene Expression Omnibus under series GSE25715.

### Small RNA deep sequencing data analysis

Color-space reads were matched against annotated databases using the Small RNA Analysis Pipeline Tool v5.0 (RNA2MAP), provided by Applied Biosystems, using the following parameters: one color-space mismatch within the first 18 bases of the reads, called the 'seed sequence' and two color-space mismatches on the following positions of the reads. First, small RNA reads were matched against the human genome (hg19), then versus miRBase release 16 to identify matches with non-human miRNA, and finally versus non-coding RNA sequences from fRNAdb, a database of ncRNA.org. For each annotated miRNA that was sequenced, the number of sequences for miRNAs was normalized to a total of 10^6 ^miRNA sequences. The amount of each miRNA was determined following a linear model. Only miRNAs with at least 300 counts per million in at least one of the experimental conditions were conserved for differential expression analysis. The significance of the difference between the experimental and control groups was estimated by an empirical Bayes method using the limma package from Bioconductor [38].

### Inactivation and over-expression of miRNAs

All transfections were performed with HighPerfect transfection reagent (Qiagen). For inactivation of miRNA expression, sub-confluent hMADS cells were transfected with a combination of three DNA/LNA mixmers with a phosphorothioate backbone (Exiqon, Vedbaek, Denmark), at a final concentration of 40 nM for each. The sequences of these three DNA/LNA mixmers were 5'-CAGTCGGGGATGTTTAC-3', 5'-CAGTCGAGGATGTTTAC-3', and 5'-GAGTGTAGGATGTTTAC-3'. The simultaneous use of these three oligonucleotides successfully inhibited all miRNAs from the miR-30 family (Exiqon, personal communication; Additional file 6). The sequence for the mismatch control oligonucleotide was CAGTCGAAGCTGTTTAC.

For over-expression, sub-confluent hMADS cells were transfected with Pre-miR™ miRNA precursor molecules (Ambion, Life Technologies Corporation, Carlsbad, CA, USA), at a final concentration of 40 nM. The negative control was the 'Pre-miR™ miRNA Precursor Molecules - Negative Control #1'.

For both over-expression and inhibition studies, hMADS cells were submitted to adipogenic medium 3 days after transfection. For the target protection experiment, sub-confluent hMADS cells were transfected with TSBs, which are custom designed LNA oligonucleotides with a phosphorothioate backbone (Exiqon); the sequences were 5'-ACATGAAGTAAACACACA-3' for miR-30-TSB and 5'-CAGTCGAAGCTGTTTAC-3' for TSB-neg (mismatch control). TSBs were used at a concentration of 20 nM. The day after this first transfection, hMADS cells were co-transfected with miR-30 Pre-miR™ miRNA precursor molecules (Ambion) at a final concentration of 40 nM, together with the miR-30-TSB again, or the mismatch control TSB. hMADS cells were then submitted to adipogenic medium the day after the second transfection.

### Cloning of *RUNX2 *3' UTR in pSi-CHECK™-2

Partial sequences (positions 32 to 332 and positions 3,102 to 3,421) from the 3' UTR of *RUNX2 *(ENST00000465038) were amplified by PCR and cloned at the XhoI and NotI sites of pSi-CHECK™-2 (Promega, Madison, WI, USA). Synthetic miRNAs (miR-30a, miR-30d and miR-378) as well as negative control (miR-Neg) were purchased from Ambion. HEK 293T cells (20,000 per well) were reverse transfected in 96-well white plates with 100 ng of pSi-CHECK™-2 plasmid and 5 nmol of synthetic miRNAs using 1 μl of lipofectamine 2000 (Invitrogen, Life Technologies Corporation, Carlsbad, CA, USA). The, 48 hours after transfection, renilla and firefly luciferase activities were assayed with the Dual Glo Luciferase Assay System (Promega) and measured with a luminometer (Luminoskan Ascent, Thermo Scientific, Waltham, MA, USA).

### Preparation of cell extracts and western blot analysis

Cells were rinsed with phosphate-buffered saline and solubilized in stop buffer containing 50 mmol/l HEPES, pH 7.2, 150 mmol/l NaCl, 10 mmol/l EDTA, 10 mmol/l Na_4_P_2_O_7_, 2 mmol/l Na_3_VO_4_, and 1% Triton X-100 supplemented with Protease Inhibitor Cocktail (Roche). RUNX2 antibody (MBL, Woburn, MA, USA) was used at a final concentration of 0.5 ng/μl. Secondary horseradish peroxidase-conjugated antibody was purchased from Promega.

## Abbreviations

bp: base pair; CBFB: core binding factor beta; C/EBP: CCAAT/enhancer-binding protein; DMEM: Dulbecco's modified Eagle's medium; FABP4: fatty acid binding protein 4; GIPR: glucose-dependent insulinotropic polypeptide receptor; GPDH: glycerol-3-phosphate dehydrogenase; hMADS: human multipotent adipose-derived stem; miRNA: microRNA; NCOA2: nuclear receptor coactivator 2; piRNA: piwi-interacting RNA; PPAR: peroxisome proliferator-activated receptor; qPCR: quantitative polymerase chain reaction; RUNX2: runt-related transcription factor 2; snoRNA: small nucleolar RNA; TIF2: transcriptional intermediary factor 2; TSB: target site blocker; UTR: untranslated region.

## Competing interests

The authors declare that they have no competing interests.

## Authors' contributions

LEZ participated in the conception and design of the study, performed experiments, analyzed and interpreted data, and drafted the manuscript. BW performed experiments and collected data. KLB analyzed data, performed statistical analysis, and reviewed the manuscript. PV performed experiments and collected data. BM conceived of the study, participated in its design and coordination and reviewed the manuscript. RW analyzed data and reviewed the manuscript. CD conceived of the study, participated in its design and coordination, collected data and reviewed the manuscript. PB conceived of the study, participated in its design and coordination, collected data and reviewed the manuscript. All authors read and approved the final manuscript.

## Supplementary Material

Additional file 1 **Figure S1**. Quantitative RT-PCR of adiponectin (AdipoQ), *PPARG2 *and *RUNX2 *in adipocyte-differentiated (day 8) versus differentiated hMADS cells. Real-time PCR was performed using LightCycler^® ^480 SYBR Green I Master mix and Light Cycler 480 real-time PCR machine (Roche Applied Science, Indianapolis, IN, USA). Expression levels of transcripts were evaluated using the comparative CT method (2-deltaCT). Transcript levels of *POLR2A *and *RPL13 *were used for sample normalization. Results are log2-transformed fold changes of normalized 2-deltaCT. Data were obtained from three independent experiments (error bars represent average ± standard error). Click here for file

Additional file 2 **Dataset showing all read count for mature miRNAs**. Click here for file

Additional file 3 **Table S1**. Summary of concordant miRNA regulation across published studies. FC, fold change; AD3, adipogenesis day 3; AD8, adipogenesis day 8. References are detailed in the references section of the main manuscript [39-42]. Click here for file

Additional file 4 **Figure S2**. Quantitative RT-PCR confirmation for eight selected miRNAs. Data represent the log2 fold-change of expression between adipocyte-differentiated (day 8) cells versus undifferentiated hMADS cells. Mature miRNA expression was evaluated using Mirscript assays (Qiagen SA, Courtaboeuf, France) as specified by the manufacturer's protocol. The forward primer for miR-642a-3p was manually designed (5'-TCGTCGAGACACATTTGGAGAG-3'). Real-time PCR was performed using LightCycler^® ^480 SYBR Green I Master mix and Light Cycler 480 real-time PCR machine (Roche Applied Science). Expression levels of mature miRNAs were evaluated using comparative the CT method (2-deltaCT). Transcript levels of *POLR2A *and *RPL13 *were used for sample normalization. Results are log2-transformed fold changes of normalized 2-deltaCT. Data were obtained from three independent experiments (error bars represent average ± standard error). Click here for file

Additional file 5 **Figure S3**. Genome-browser representation of reads matching **(a) **miR-642a and **(b) **miR-378. For each nucleotide, the corresponding read count was printed. For each panel, the following information is represented, from top to bottom: chromosomal location, genomic coordinates, counts for each sample (on the plus and minus strand) and transcripts annotations (RefSeq Genes). Read counts correspond to undifferentiated (ND.1) and day 8 differentiated (AD8.1) hMADS cells samples. Only counts from the first biological replicate, with a reading from 3' to 5', are represented. Click here for file

Additional file 6 **Figure S4**. Quantitative RT-PCR confirmation of inhibition or over-expression of the miR-30 family. Sub-confluent hMADS cells were transfected and induced to differentiate as described in Material and methods, 3 days after transfection. **(a) **For inhibition of the miR-30 family, RNA was extracted and analyzed at day 10 of differentiation. **(b) **For over-expression of pre-miR30a and pre-miR-30d, RNA was extracted and analyzed at day 4 of differentiation. Mature miRNA expression was evaluated using Mirscript assays (Qiagen SA) as specified by the manufacturer's protocol. Real-time PCR was performed using LightCycler^® ^480 SYBR Green I Master mix and Light Cycler 480 real-time PCR machine (Roche Applied Science). Expression levels of mature miRNAs were evaluated using the comparative CT method (2-deltaCT). Transcript levels of *POLR2A *and *TBP *were used for sample normalization. Results are log2-transformed fold changes of normalized 2-deltaCT. Data were obtained from three independent experiments (error bars represent average ± standard error). Click here for file

Additional file 7 **Figure S5**. Screen shot from TargetScan (release 5.1) showing conserved and poorly conserved miR-30 family putative binding sites located in the 3' UTR of human *RUNX2*. Click here for file

Additional file 8 **Tables S2 and S3**. Table S2: miR-30 family identifiers, genomic coordinates and mature sequences. Grey shading indicates identical sequences. Table S3: miR-642 family identifiers, genomic coordinates and mature sequences. Grey shading indicates identical sequences. Click here for file

Additional file 9 **Table S4 and Figure S6**. Table S4: miR-642 raw read numbers. AD8, adipogenesis day 8. Figure S6: Quality values according to base position along miR-642-3p reads. Top panel: values for sequencing from the 5' to 3' end. Bottom panel: values for sequencing from the 3' to 5' end. Bases that allow discrimination between miR-642-3p and miR-642b are highlighted in blue and indicated by arrows. Click here for file

Additional file 10 **Figures S7 and S8**. Figure S7: genome-browser representation of reads matching the fourth intron of NCOA2. For each nucleotide, the corresponding read count was printed. The following information is represented, from top to bottom: chromosomal location, genomic coordinates, counts for each sample, transcript annotations (RefSeq Genes) and non-coding RNA annotations (ncRNA.org). Read counts correspond to undifferentiated (ND.1 and ND.2), day 3 differentiated (AD3.1 and AD3.2) and day 8 differentiated (AD8.1 and AD8.2) hMADS cell samples, with sequencing from the 3' to 5' end. Figure S8: genome browser representation of reads matching **(a) **tRNA32.LysCTT and **(b) **tRNA113.AlaTGC. For each nucleotide, the corresponding read count was printed. For each panel, the following information is represented, from top to bottom: chromosomal location, genomic coordinates, counts for each sample, transcript annotations (RefSeq Genes) and non-coding RNA annotations (ncRNA.org). Read counts correspond to undifferentiated (ND.1 and ND.2), day 3 differentiated (AD3.1 and AD3.2) and day 8 differentiated (AD8.1 and AD8.2) hMADS cell samples, with sequencing from the 3' to 5' end. Click here for file

Additional file 11 **Table S5**. PCR primer sequences. Click here for file
