# Supplementary material for: Small RNA sequencing reveals miR-642a-3p as a novel adipocyte-specific microRNA and miR-30 as a key regulator of human adipogenesis
Source: Genome Biol. 2011 Jul 18;12(7):R64. doi: 10.1186/gb-2011-12-7-r64 (PMC3218826; doi:10.1186/gb-2011-12-7-r64)
Supplement: Additional file 1 — Figure S1. Quantitative RT-PCR of adiponectin (AdipoQ), PPARG2 and RUNX2 in adipocyte-differentiated (day 8) versus differentiated hMADS cells. Real-time PCR was performed using LightCycler® 480 SYBR Green I Master mix and Light Cycler 480 real-time PCR machine (Roche Applied Science, Indianapolis, IN, USA). Expression levels of transcripts were evaluated using the comparative CT method (2-deltaCT). Transcript levels of POLR2A and RPL13 were used for sample normalization. Results are log2-transformed fold changes of normalized 2-deltaCT. Data were obtained from three independent experiments (error bars represent average ± standard error). [file gb-2011-12-7-r64-S1.PDF]

Additional File 1

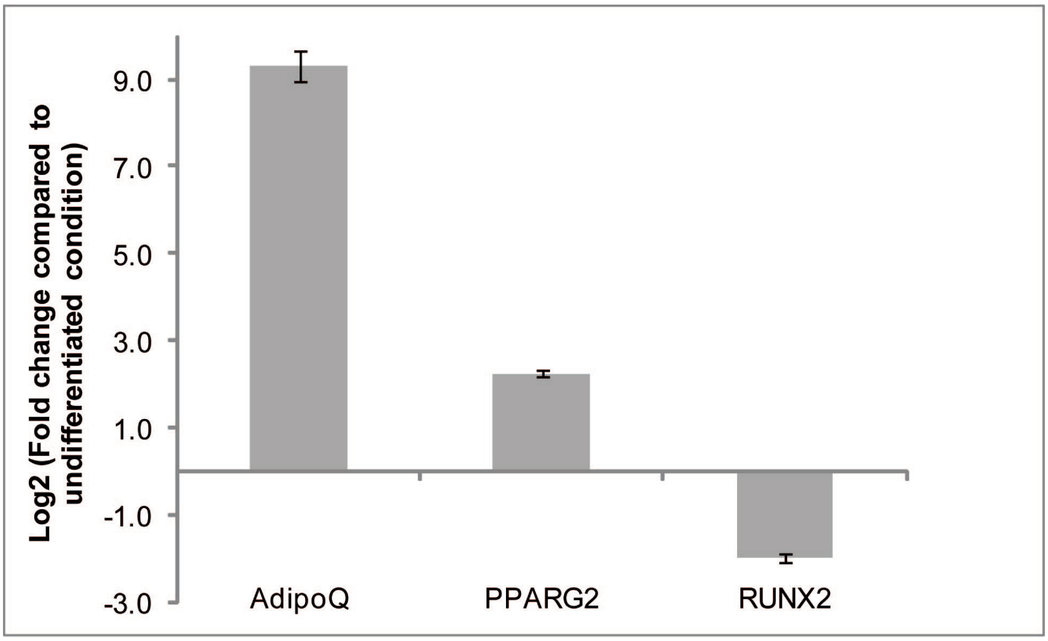

Figure S1: Quantitative RT-PCR of adiponectin (AdipoQ), PPARG2 and RUNX2 in adipocyte-differentiated (day 8) vs. differentiated hMADS cells

Real-time PCR was performed using LightCycler® 480 SYBR Green I Master mix and Light Cycler 480 real-time PCR machine (Roche Applied Science, Indianapolis, USA). Expression levels of transcripts were evaluated using comparative CT method (2-deltaCT). Transcript levels of POLR2A and RPL13 were used for sample normalization. Results are log2-transformed fold changes of normalized 2-deltaCT. Data was obtained from 3 independent experiments (Bars: average ± se).
