# Supplementary material for: Small RNA sequencing reveals miR-642a-3p as a novel adipocyte-specific microRNA and miR-30 as a key regulator of human adipogenesis
Source: Genome Biol. 2011 Jul 18;12(7):R64. doi: 10.1186/gb-2011-12-7-r64 (PMC3218826; doi:10.1186/gb-2011-12-7-r64)
Supplement: Additional file 3 — Table S1. Summary of concordant miRNA regulation across published studies. FC, fold change; AD3, adipogenesis day 3; AD8, adipogenesis day 8. References are detailed in the references section of the main manuscript [39-42]. [file gb-2011-12-7-r64-S3.PDF]

Additional File 3

<u>Table S1:</u> Summary of concordant miRNA regulation across studies. FC, fold change; AD3, adipogenesis day 3; AD8, adipogenesis day 8. References are detailed in the references section of the main manuscript.

miRNA	FC AD3[a]	FC AD8[b]	Reference	cell models	Profiling technique
miR-34b*	4.1	4.51	[39]	human preadipocytes	Microarray
miR-378[†]	0.73	4.33	[39]<br>[40]<br>[14]<br>[30]	3T3-L1 (mouse preadipocytes), human preadipocytes	Microarray<br>Cloning/capillary sequencing<br>Quantitative PCR
miRNA-378*[†]	0.28	4.24	[30]	3T3-L1	Quantitative PCR
miR-34a	3.7	3.74	[39]	human preadipocytes	Microarray
miR-101	1.02	3.48	[39]<br>[41]	3T3-L1	Microarray
miR-92a	2.15	3.14	[16]<br>[15]	3T3-L1, human preadipocytes	Microarray
miR-186	0.36	2.15	[39]	human preadipocytes	Microarray
miR-30a<br>miR-30b<br>miR-30c<br>miR-30e	1.55<br>0.52<br>1.09<br>0.53	3.06<br>1.82<br>2.49<br>1.41	[39]<br>[15]<br>[42]<br>[14]	3T3-L1, human preadipocytes, human bone marrow mesenchymal stem cells	Microarray<br>Bead-based flow cytometric method
miR-10b	0.56	1.25	[41]<br>[42]	3T3-L1, human bone marrow mesenchymal stem cells	Microarray<br>Bead-based flow cytometric method
let-7e	-0.85	-1.48	[41]	3T3-L1	Microarray

[a] Fold-Change (log2) observed between partially adipocyte-differentiated cells (day 3) and undifferentiated cells in the present study

[b] Fold-Change (log2) observed between terminally adipocyte-differentiated cells (day 8) and undifferentiated cells in the present study

[†] Previously termed mir-422b in mirBase versions prior to release 10.0
