# Supplementary material for: Small RNA sequencing reveals miR-642a-3p as a novel adipocyte-specific microRNA and miR-30 as a key regulator of human adipogenesis
Source: Genome Biol. 2011 Jul 18;12(7):R64. doi: 10.1186/gb-2011-12-7-r64 (PMC3218826; doi:10.1186/gb-2011-12-7-r64)
Supplement: Additional file 4 — Figure S2. Quantitative RT-PCR confirmation for eight selected miRNAs. Data represent the log2 fold-change of expression between adipocyte-differentiated (day 8) cells versus undifferentiated hMADS cells. Mature miRNA expression was evaluated using Mirscript assays (Qiagen SA, Courtaboeuf, France) as specified by the manufacturer's protocol. The forward primer for miR-642a-3p was manually designed (5'-TCGTCGAGACACATTTGGAGAG-3'). Real-time PCR was performed using LightCycler® 480 SYBR Green I Master mix and Light Cycler 480 real-time PCR machine (Roche Applied Science). Expression levels of mature miRNAs were evaluated using comparative the CT method (2-deltaCT). Transcript levels of POLR2A and RPL13 were used for sample normalization. Results are log2-transformed fold changes of normalized 2-deltaCT. Data were obtained from three independent experiments (error bars represent average ± standard error). [file gb-2011-12-7-r64-S4.PDF]

Additional File 4

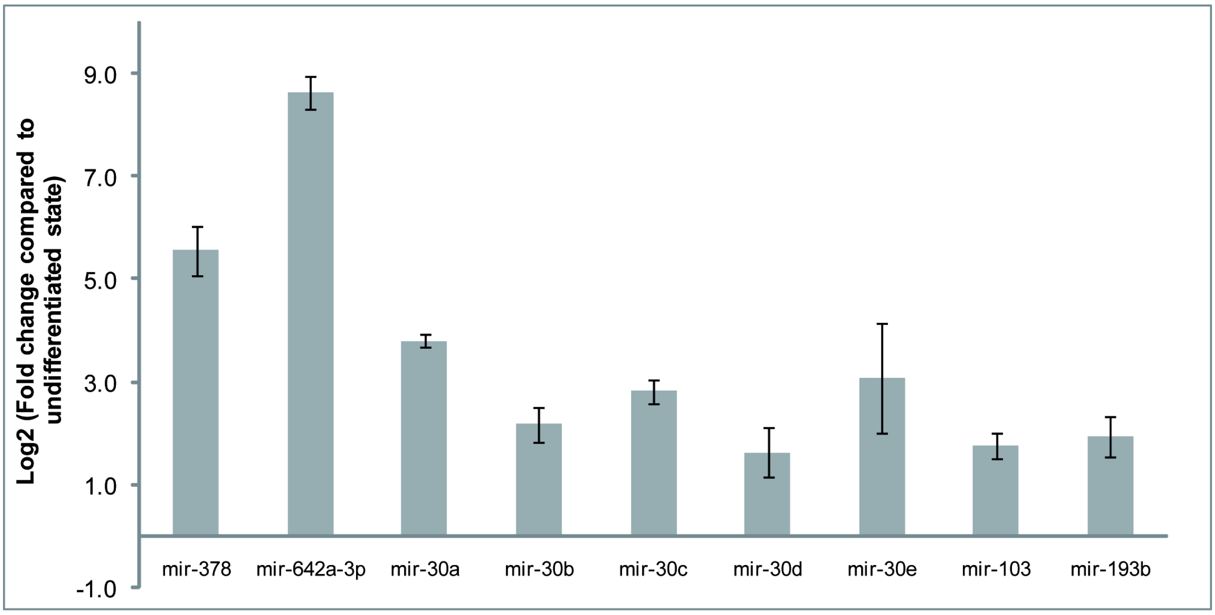

<u>Figure S2:</u> Quantitative RT-PCR confirmation for 8 selected miRNAs

Data represent the log2 fold-change of expression between adipocyte-differentiated (day 8) cells vs. undifferentiated cells. Mature microRNA expression was evaluated using Mirscript assays (Qiagen SA, Courtaboeuf, France) as specified by the manufacturer's protocol. Forward primer for miR-642a-3p was manually designed (5'-TCGTCGAGACACATTTGGAGAG-3'). Real-time PCR was performed using LightCycler® 480 SYBR Green I Master mix and Light Cycler 480 real-time PCR machine (Roche Applied Science, Indianapolis, United-States). Expression levels of mature microRNAs were evaluated using comparative CT method (2-deltaCT). Transcript levels of POLR2A and RPL13 were used for sample normalization. Results are log2-transformed fold changes of normalized 2-deltaCT. Data was obtained from 3 independent experiments (Bars: average ± se).
