# Supplementary material for: Small RNA sequencing reveals miR-642a-3p as a novel adipocyte-specific microRNA and miR-30 as a key regulator of human adipogenesis
Source: Genome Biol. 2011 Jul 18;12(7):R64. doi: 10.1186/gb-2011-12-7-r64 (PMC3218826; doi:10.1186/gb-2011-12-7-r64)
Supplement: Additional file 5 — Figure S3. Genome-browser representation of reads matching (a) miR-642a and (b) miR-378. For each nucleotide, the corresponding read count was printed. For each panel, the following information is represented, from top to bottom: chromosomal location, genomic coordinates, counts for each sample (on the plus and minus strand) and transcripts annotations (RefSeq Genes). Read counts correspond to undifferentiated (ND.1) and day 8 differentiated (AD8.1) hMADS cells samples. Only counts from the first biological replicate, with a reading from 3' to 5', are represented. [file gb-2011-12-7-r64-S5.PDF]

Additional File 5

A

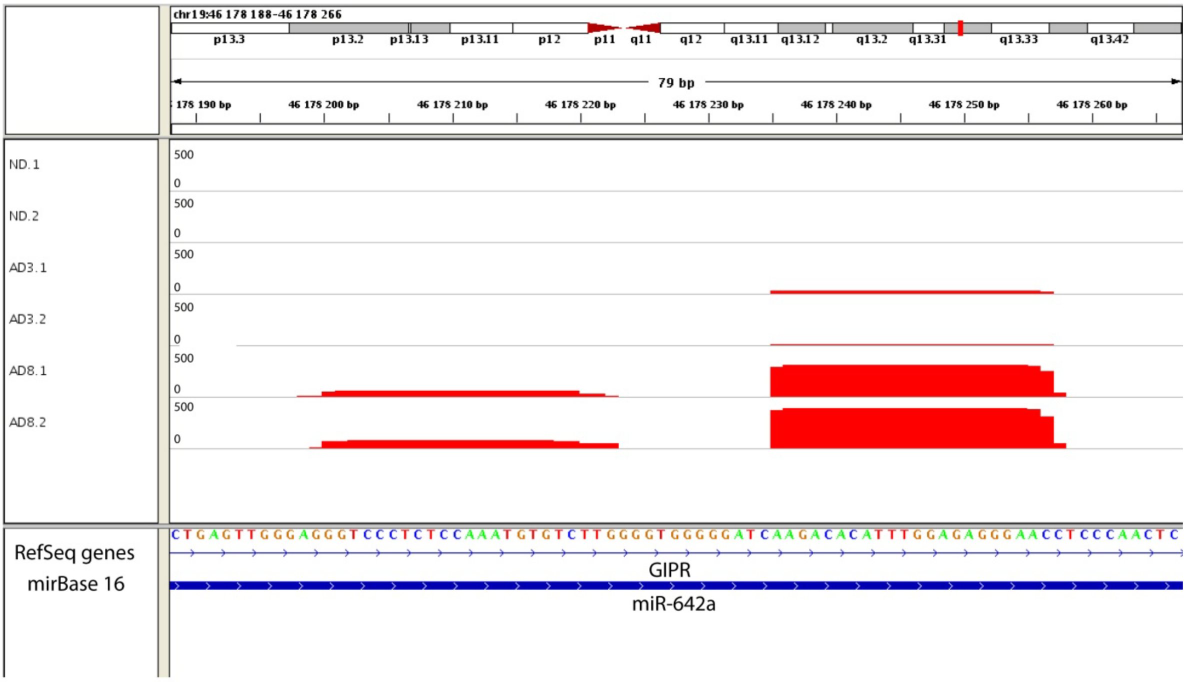

B

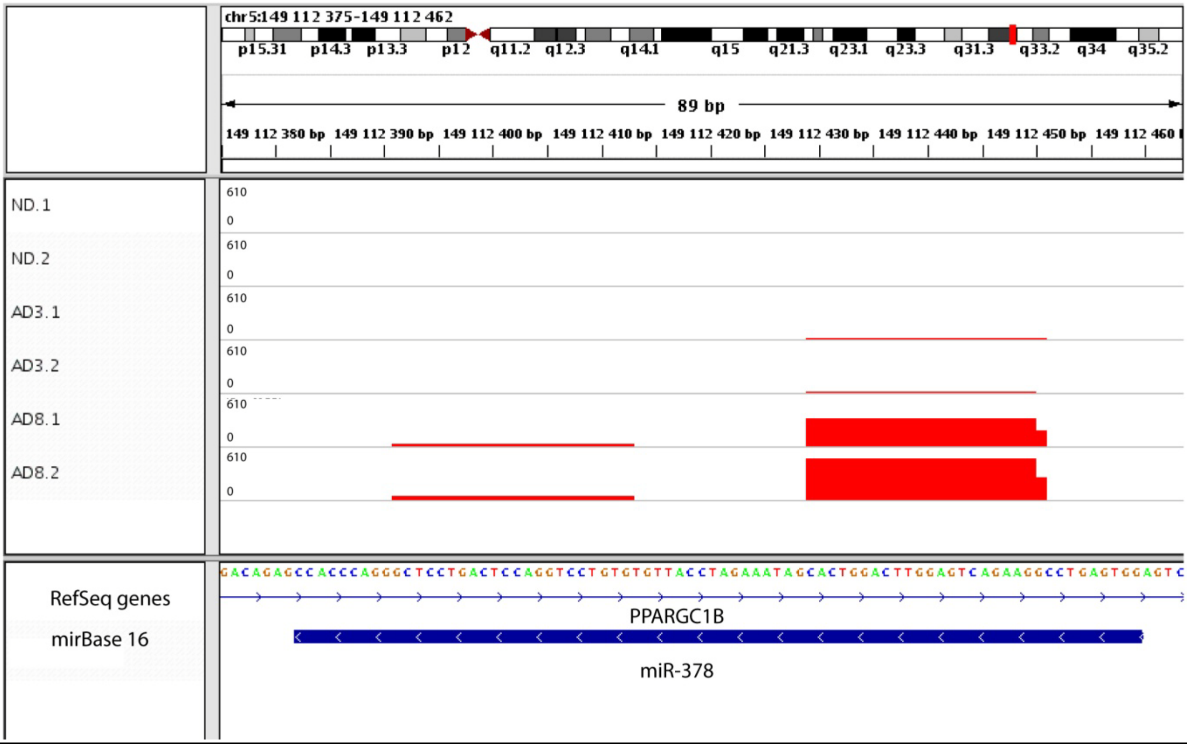

<u>Figure S3:</u> Genome-browser representation of reads matching miR-642a (A) and miR-378 (B)

For each nucleotide, the corresponding read count was printed. For each panel, the following information is represented, from top to bottom: chromosomal location, genomic coordinates, the counts for each sample (on the plus and minus strand) and transcripts annotations (RefSeq Genes). Read counts correspond to undifferentiated (ND.1) and day-8 differentiated (AD8.1) hMADS cells samples. Only counts from the first biological replicate, with a reading from 3' to 5' are represented.
