# Supplementary material for: Small RNA sequencing reveals miR-642a-3p as a novel adipocyte-specific microRNA and miR-30 as a key regulator of human adipogenesis
Source: Genome Biol. 2011 Jul 18;12(7):R64. doi: 10.1186/gb-2011-12-7-r64 (PMC3218826; doi:10.1186/gb-2011-12-7-r64)
Supplement: Additional file 6 — Figure S4. Quantitative RT-PCR confirmation of inhibition or over-expression of the miR-30 family. Sub-confluent hMADS cells were transfected and induced to differentiate as described in Material and methods, 3 days after transfection. (a) For inhibition of the miR-30 family, RNA was extracted and analyzed at day 10 of differentiation. (b) For over-expression of pre-miR30a and pre-miR-30d, RNA was extracted and analyzed at day 4 of differentiation. Mature miRNA expression was evaluated using Mirscript assays (Qiagen SA) as specified by the manufacturer's protocol. Real-time PCR was performed using LightCycler® 480 SYBR Green I Master mix and Light Cycler 480 real-time PCR machine (Roche Applied Science). Expression levels of mature miRNAs were evaluated using the comparative CT method (2-deltaCT). Transcript levels of POLR2A and TBP were used for sample normalization. Results are log2-transformed fold changes of normalized 2-deltaCT. Data were obtained from three independent experiments (error bars represent average ± standard error). [file gb-2011-12-7-r64-S6.PDF]

Additional File 6

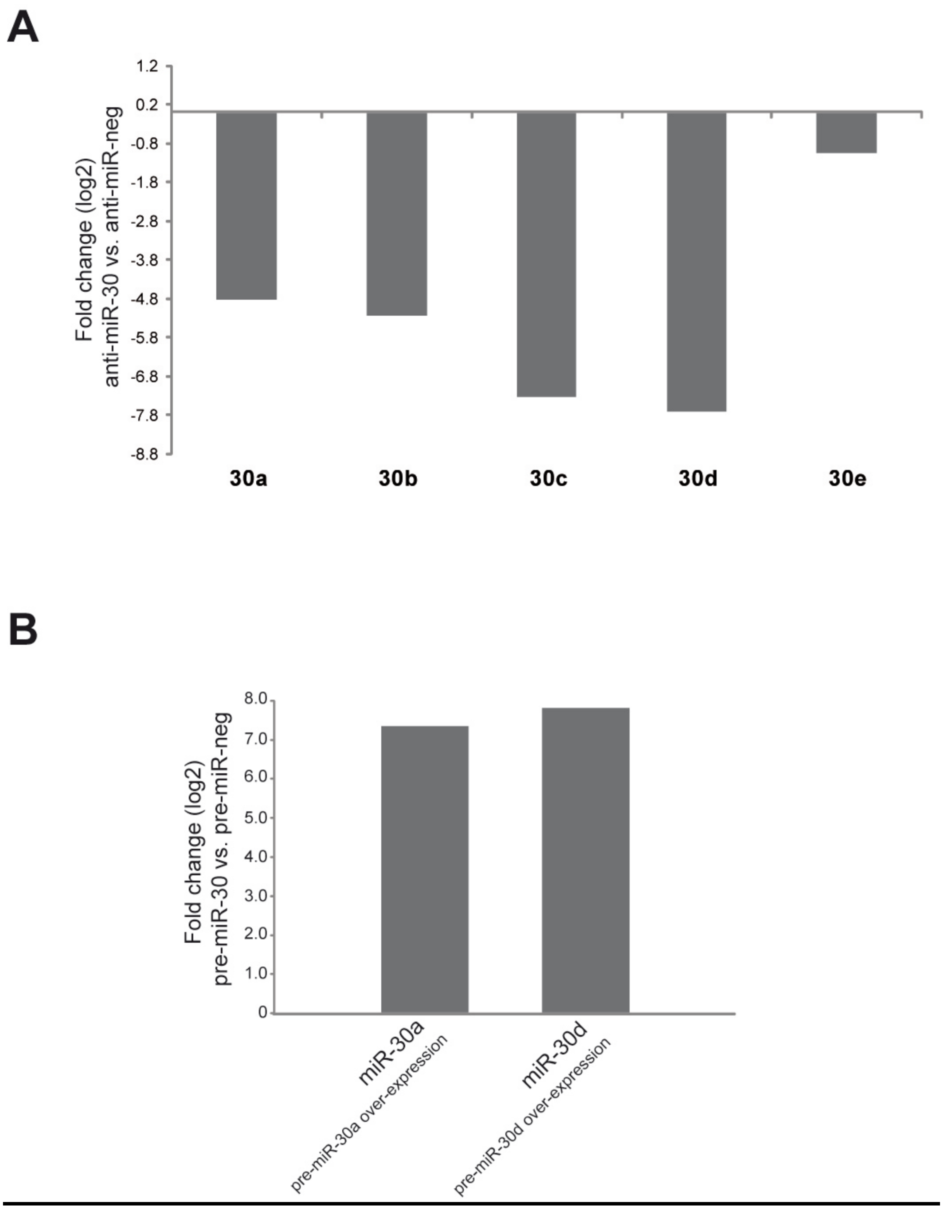

<u>Figure S4 :</u> Quantitative RT-PCR confirmation of inhibition or over-expression of the miR-30 family

Sub-confluent hMADS cells were transfected and induced to differentiate as described in "material and methods", 3 days after transfection. For inhibition of the miR-30 family **(A)**, RNA was extracted and analyzed at day 10 of differentiation. For over-expression of pre-miR30a and -30d **(B)**, RNA was extracted and analyzed at day 4 of differentiation. Mature

microRNA expression was evaluated using Mirscript assays (Qiagen SA, Courtaboeuf, France) as specified by the manufacturer's protocol. Real-time PCR was performed using LightCycler® 480 SYBR Green I Master mix and Light Cycler 480 real-time PCR machine (Roche Applied Science, Indianapolis, USA). Expression levels of mature microRNAs were evaluated using comparative CT method (2-deltaCT). Transcript levels of POLR2A and TBP were used for sample normalization. Results are log2-transformed fold changes of normalized 2-deltaCT. Data was obtained from 3 independent experiments (Bars: average ± se).
