# Supplementary material for: Small RNA sequencing reveals miR-642a-3p as a novel adipocyte-specific microRNA and miR-30 as a key regulator of human adipogenesis
Source: Genome Biol. 2011 Jul 18;12(7):R64. doi: 10.1186/gb-2011-12-7-r64 (PMC3218826; doi:10.1186/gb-2011-12-7-r64)
Supplement: Additional file 7 — Figure S5. Screen shot from TargetScan (release 5.1) showing conserved and poorly conserved miR-30 family putative binding sites located in the 3' UTR of human RUNX2. [file gb-2011-12-7-r64-S7.PDF]

Additional File 7

Conserved

<table>
<tr><th></th><th>predicted consequential pairing of target region (top) and miRNA (bottom)</th><th>seed match</th><th>site-type contri-bution</th><th>3' pairing contri-bution</th><th>local AU contri-bution</th><th>position contri-bution</th><th>context score</th><th>context score percentile</th><th>conserved branch length</th><th>P_{CT}</th></tr>
<tr><td>Position 3348-3354 of RUNX2 3' UTR
hsa-miR-30b</td><td>5' ...AAUUUUUUAGAUGUGUGUUUACU...
|||||||
3' UCGACUCACAUCCUACAAAUGU</td><td>7mer-m8</td><td>-0.161</td><td>-0.011</td><td>-0.065</td><td>0.001</td><td>-0.23</td><td>60</td><td>1.317</td><td>0.38</td></tr>
<tr><td>Position 3348-3354 of RUNX2 3' UTR
hsa-miR-30c</td><td>5' ...AAUUUUUUAGAUGUGUGUUUACU...
|||||||
3' CGACUCUCACAUCCUACAAAUGU</td><td>7mer-m8</td><td>-0.161</td><td>-0.011</td><td>-0.065</td><td>0.001</td><td>-0.23</td><td>59</td><td>1.317</td><td>0.38</td></tr>
<tr><td>Position 3348-3354 of RUNX2 3' UTR
hsa-miR-30d</td><td>5' ...AAUUUUUUAGAUGUGUGUUUACU...
|||||||
3' GAAGGUCAGCCCCUACAAAUGU</td><td>7mer-m8</td><td>-0.161</td><td>0.020</td><td>-0.065</td><td>0.001</td><td>-0.20</td><td>54</td><td>1.317</td><td>0.38</td></tr>
<tr><td>Position 3348-3354 of RUNX2 3' UTR
hsa-miR-30a</td><td>5' ...AAUUUUUUAGAUGUGUGUUUACU...
|||||||
3' GAAGGUCAGCUCCUACAAAUGU</td><td>7mer-m8</td><td>-0.161</td><td>0.020</td><td>-0.065</td><td>0.001</td><td>-0.20</td><td>53</td><td>1.317</td><td>0.38</td></tr>
<tr><td>Position 3348-3354 of RUNX2 3' UTR
hsa-miR-30e</td><td>5' ...AAUUUUUUAGAUGUGUGUUUACU...
|||||||
3' GAAGGUCAGUUCCUACAAAUGU</td><td>7mer-m8</td><td>-0.161</td><td>0.020</td><td>-0.065</td><td>0.001</td><td>-0.20</td><td>51</td><td>1.317</td><td>0.38</td></tr>
<tr><td>Position 3359-3365 of RUNX2 3' UTR
hsa-miR-30a</td><td>5' ...UGUGUGUUUACUUCAUGUUUACA...
|||||||
3' GAAGGUCAGCUCCUACAAAUGU</td><td>8mer</td><td>-0.310</td><td>0.003</td><td>-0.129</td><td>0.001</td><td>-0.43</td><td>93</td><td>1.409</td><td>0.54</td></tr>
<tr><td>Position 3359-3365 of RUNX2 3' UTR
hsa-miR-30b</td><td>5' ...UGUGUGUUUACUUCAUGUUUACA...
|||| |||||||
3' UCGACUCACAUCCU----ACAAAUGU</td><td>8mer</td><td>-0.310</td><td>-0.001</td><td>-0.129</td><td>0.001</td><td>-0.43</td><td>93</td><td>1.409</td><td>0.54</td></tr>
<tr><td>Position 3359-3365 of RUNX2 3' UTR
hsa-miR-30e</td><td>5' ...UGUGUGUUUACUUCAUGUUUACA...
|||||||
3' GAAGGUCAGUUCCUACAAAUGU</td><td>8mer</td><td>-0.310</td><td>0.003</td><td>-0.129</td><td>0.001</td><td>-0.43</td><td>93</td><td>1.409</td><td>0.54</td></tr>
<tr><td>Position 3359-3365 of RUNX2 3' UTR
hsa-miR-30d</td><td>5' ...UGUGUGUUUACUUCAUGUUUACA...
|||||||
3' GAAGGUCAGCCCCUACAAAUGU</td><td>8mer</td><td>-0.310</td><td>0.003</td><td>-0.129</td><td>0.001</td><td>-0.43</td><td>93</td><td>1.409</td><td>0.54</td></tr>
<tr><td>Position 3359-3365 of RUNX2 3' UTR
hsa-miR-30c</td><td>5' ...UGUGUGUUUACUUCAUGUUUACA...
|||| |||||||
3' CGACUCUCACAUCCU----ACAAAUGU</td><td>8mer</td><td>-0.310</td><td>-0.001</td><td>-0.129</td><td>0.001</td><td>-0.43</td><td>93</td><td>1.409</td><td>0.54</td></tr>
</table>

Poorly conserved

<table>
<tr><th></th><th>predicted consequential pairing of target region (top) and miRNA (bottom)</th><th>seed match</th><th>site-type contri-bution</th><th>3' pairing contri-bution</th><th>local AU contri-bution</th><th>position contri-bution</th><th>context score</th><th>context score percentile</th><th>conserved branch length</th><th>P_{CT}</th></tr>
<tr><td>Position 229-235 of RUNX2 3' UTR
hsa-miR-30e</td><td>5' ...AAUCGAGCUUCAGAU-UGUUUACU...
||| |||||||
3' GAAGGUCAGUUCCUACAAAUGU</td><td>7mer-m8</td><td>-0.161</td><td>-0.026</td><td>-0.119</td><td>-0.016</td><td>-0.32</td><td>79</td><td>0.286</td><td>< 0.1</td></tr>
<tr><td>Position 229-235 of RUNX2 3' UTR
hsa-miR-30d</td><td>5' ...AAUCGAGCUUCAGAUUGUUUACU...
|||||||
3' GAAGGUCAGCCCCUACAAAUGU</td><td>7mer-m8</td><td>-0.161</td><td>0.005</td><td>-0.119</td><td>-0.016</td><td>-0.29</td><td>74</td><td>0.286</td><td>< 0.1</td></tr>
<tr><td>Position 229-235 of RUNX2 3' UTR
hsa-miR-30a</td><td>5' ...AAUCGAGCUUCAGAUUGUUUACU...
|||||||
3' GAAGGUCAGCUCCUACAAAUGU</td><td>7mer-m8</td><td>-0.161</td><td>0.005</td><td>-0.119</td><td>-0.016</td><td>-0.29</td><td>74</td><td>0.286</td><td>< 0.1</td></tr>
<tr><td>Position 229-235 of RUNX2 3' UTR
hsa-miR-30b</td><td>5' ...AAUCGAGCUUCAGAUUGUUUACU...
|||||||
3' UCGACUCACAUCCUACAAAUGU</td><td>7mer-m8</td><td>-0.161</td><td>0.005</td><td>-0.119</td><td>-0.016</td><td>-0.29</td><td>73</td><td>0.286</td><td>< 0.1</td></tr>
<tr><td>Position 229-235 of RUNX2 3' UTR
hsa-miR-30c</td><td>5' ...AAUCGAGCUUCAGAUUGUUUACU...
|||||||
3' CGACUCUCACAUCCUACAAAUGU</td><td>7mer-m8</td><td>-0.161</td><td>0.005</td><td>-0.119</td><td>-0.016</td><td>-0.29</td><td>73</td><td>0.286</td><td>< 0.1</td></tr>
<tr><td>Position 2463-2469 of RUNX2 3' UTR
hsa-miR-30c</td><td>5' ...AAAGGGCUUUUUUAAGUUUACAG...
||||||
3' CGACUCUCACAUCCUACAAAUGU</td><td>7mer-1A</td><td>-0.099</td><td>0.025</td><td>-0.052</td><td>0.062</td><td>-0.06</td><td>9</td><td>0.921</td><td>< 0.1</td></tr>
<tr><td>Position 2463-2469 of RUNX2 3' UTR
hsa-miR-30b</td><td>5' ...AAAGGGCUUUUUUAAGUUUACAG...
||||||
3' UCGACUCACAUCCUACAAAUGU</td><td>7mer-1A</td><td>-0.099</td><td>0.025</td><td>-0.052</td><td>0.062</td><td>-0.06</td><td>9</td><td>0.921</td><td>< 0.1</td></tr>
<tr><td>Position 2463-2469 of RUNX2 3' UTR
hsa-miR-30e</td><td>5' ...AAAGGGCUUUUUUAAGUUUACAG...
||||||
3' GAAGGUCAGUUCCUACAAAUGU</td><td>7mer-1A</td><td>-0.099</td><td>0.025</td><td>-0.052</td><td>0.062</td><td>-0.06</td><td>9</td><td>0.921</td><td>< 0.1</td></tr>
<tr><td>Position 2463-2469 of RUNX2 3' UTR
hsa-miR-30a</td><td>5' ...AAAGGGCUUUUUUAAGUUUACAG...
||||||
3' GAAGGUCAGCUCCUACAAAUGU</td><td>7mer-1A</td><td>-0.099</td><td>0.025</td><td>-0.052</td><td>0.062</td><td>-0.06</td><td>9</td><td>0.921</td><td>< 0.1</td></tr>
<tr><td>Position 2463-2469 of RUNX2 3' UTR
hsa-miR-30d</td><td>5' ...AAAGGGCUUUUUUAAGUUUACAG...
||||||
3' GAAGGUCAGCCCCUACAAAUGU</td><td>7mer-1A</td><td>-0.099</td><td>0.025</td><td>-0.052</td><td>0.062</td><td>-0.06</td><td>9</td><td>0.921</td><td>< 0.1</td></tr>
</table>

Figure S5: Screen shot from http://www.targetscan.org/ (release 5.1) showing conserved and poorly conserved miR-30 family putative binding sites which are located in human RUNX2 3'-UTR.
