# Supplementary material for: Small RNA sequencing reveals miR-642a-3p as a novel adipocyte-specific microRNA and miR-30 as a key regulator of human adipogenesis
Source: Genome Biol. 2011 Jul 18;12(7):R64. doi: 10.1186/gb-2011-12-7-r64 (PMC3218826; doi:10.1186/gb-2011-12-7-r64)
Supplement: Additional file 8 — Tables S2 and S3. Table S2: miR-30 family identifiers, genomic coordinates and mature sequences. Grey shading indicates identical sequences. Table S3: miR-642 family identifiers, genomic coordinates and mature sequences. Grey shading indicates identical sequences. [file gb-2011-12-7-r64-S8.PDF]

Additional File 8

Table S2: MiR-30 family identifiers, genomic coordinates and mature sequences. Grey shading indicate identical sequences

Mature miRNA identifier	MirBase accession number	Genomic coordinates	Strand	Mature miR sequence
**hsa-mir-30a**	MI0000088	6:72169975-72170045	-	UGUAAACAUCCUCGACUGGAAG
**hsa-mir-30b**	MI0000441	8:135881945-135882032	-	UGUAAACAUCCUACACUCAGCU
**hsa-mir-30c-1**	MI0000736	1:40995543-40995631	+	UGUAAACAUCCUACACUCUCAGC
**hsa-mir30c-2**	MI0000254	6:72143384-72143455	-	UGUAAACAUCCUACACUCUCAGC
**hsa-mir-30d**	MI0000255	8:135886301-135886370	-	UGUAAACAUCCCCGACUGGAAG
**hsa-mir-30e**	MI0000749	1:40992614-40992705	+	UGUAAACAUCCUUGACUGGAAG

Table S3: MiR-642 family identifiers, genomic coordinates and mature sequences. Grey shading indicate identical sequences

Mature miRNA identifier	MirBase accession number	Genomic coordinates	Strand	Mature miR sequence
**hsa-miR-642a (5p)**	MI0003657	19: 46178186-46178282	+	GUCCCUCUCCAAAUGUGUCUUG
**hsa-miR-642a-3p†**	†	19: 46178186-46178282	+	AGACACAUUUGGAGAGGGAACC
**hsa-miR-642b (3p)**	MI0016685	19: 46178190-46178266	-	AGACACAUUUGGAGAGGGACCC

† not yet annotated in mirBase.
