# Supplementary material for: Small RNA sequencing reveals miR-642a-3p as a novel adipocyte-specific microRNA and miR-30 as a key regulator of human adipogenesis
Source: Genome Biol. 2011 Jul 18;12(7):R64. doi: 10.1186/gb-2011-12-7-r64 (PMC3218826; doi:10.1186/gb-2011-12-7-r64)
Supplement: Additional file 9 — Table S4 and Figure S6. Table S4: miR-642 raw read numbers. AD8, adipogenesis day 8. Figure S6: Quality values according to base position along miR-642-3p reads. Top panel: values for sequencing from the 5' to 3' end. Bottom panel: values for sequencing from the 3' to 5' end. Bases that allow discrimination between miR-642-3p and miR-642b are highlighted in blue and indicated by arrows. [file gb-2011-12-7-r64-S9.PDF]

Additional File 9

<u>Table S4:</u> MiR-642 raw read numbers. AD8, adipogenesis day 8.

mature miR identifier	color space sequence (3' to 5' sequencing)	Raw read number in AD8_1	Raw read number in AD8_2
**miR-642a-5p**	1022111130010222220021	52	83
**miR-642a-3p**	10200222201003111122	445	594
**miR-642b**	0012002222010031111122	11	10

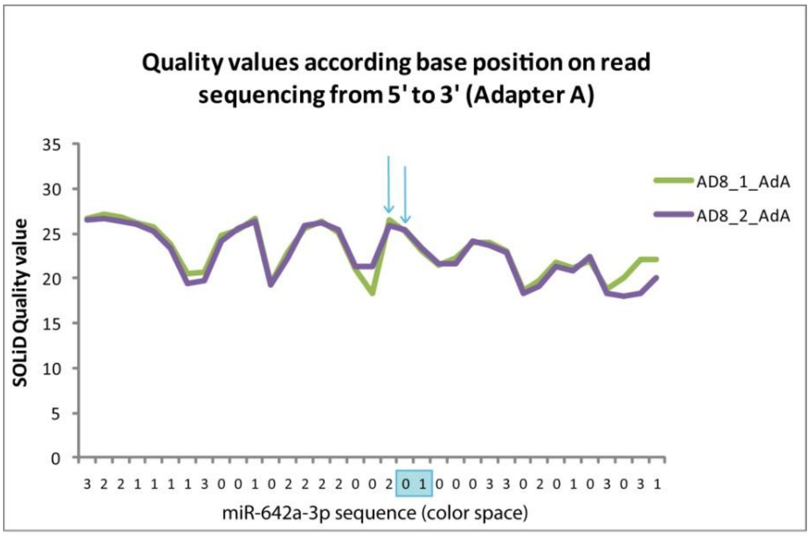

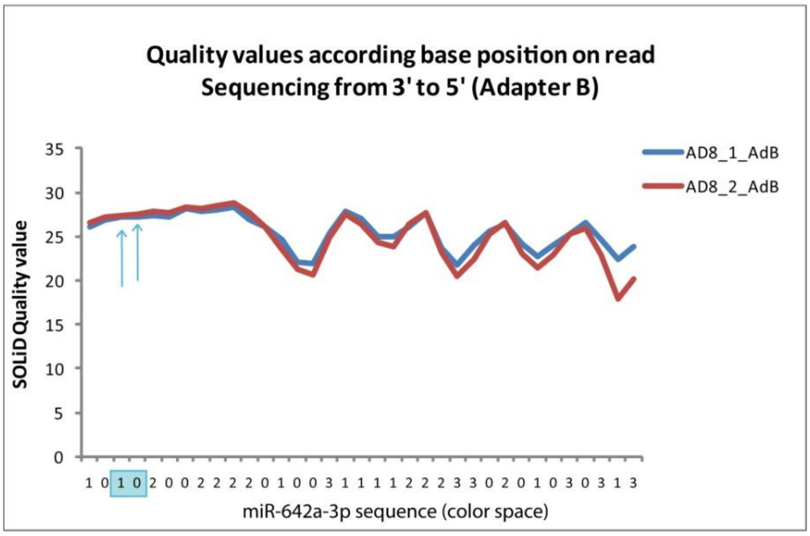

<u>Figure S6:</u> Quality values according to base position along miR-642-3p reads

Top panel: values for sequencing from the 5’ to 3’ end. **Bottom panel**: values for sequencing from the 3’ to 5’ end. Bases that allow discrimination between miR-642-3p and miR-642b are highlighted in blue, and pointed by arrows.
