# Supplementary material for: Small RNA sequencing reveals miR-642a-3p as a novel adipocyte-specific microRNA and miR-30 as a key regulator of human adipogenesis
Source: Genome Biol. 2011 Jul 18;12(7):R64. doi: 10.1186/gb-2011-12-7-r64 (PMC3218826; doi:10.1186/gb-2011-12-7-r64)
Supplement: Additional file 10 — Figures S7 and S8. Figure S7: genome-browser representation of reads matching the fourth intron of NCOA2. For each nucleotide, the corresponding read count was printed. The following information is represented, from top to bottom: chromosomal location, genomic coordinates, counts for each sample, transcript annotations (RefSeq Genes) and non-coding RNA annotations (ncRNA.org). Read counts correspond to undifferentiated (ND.1 and ND.2), day 3 differentiated (AD3.1 and AD3.2) and day 8 differentiated (AD8.1 and AD8.2) hMADS cell samples, with sequencing from the 3' to 5' end. Figure S8: genome browser representation of reads matching (a) tRNA32.LysCTT and (b) tRNA113.AlaTGC. For each nucleotide, the corresponding read count was printed. For each panel, the following information is represented, from top to bottom: chromosomal location, genomic coordinates, counts for each sample, transcript annotations (RefSeq Genes) and non-coding RNA annotations (ncRNA.org). Read counts correspond to undifferentiated (ND.1 and ND.2), day 3 differentiated (AD3.1 and AD3.2) and day 8 differentiated (AD8.1 and AD8.2) hMADS cell samples, with sequencing from the 3' to 5' end. [file gb-2011-12-7-r64-S10.PDF]

Additional File 10

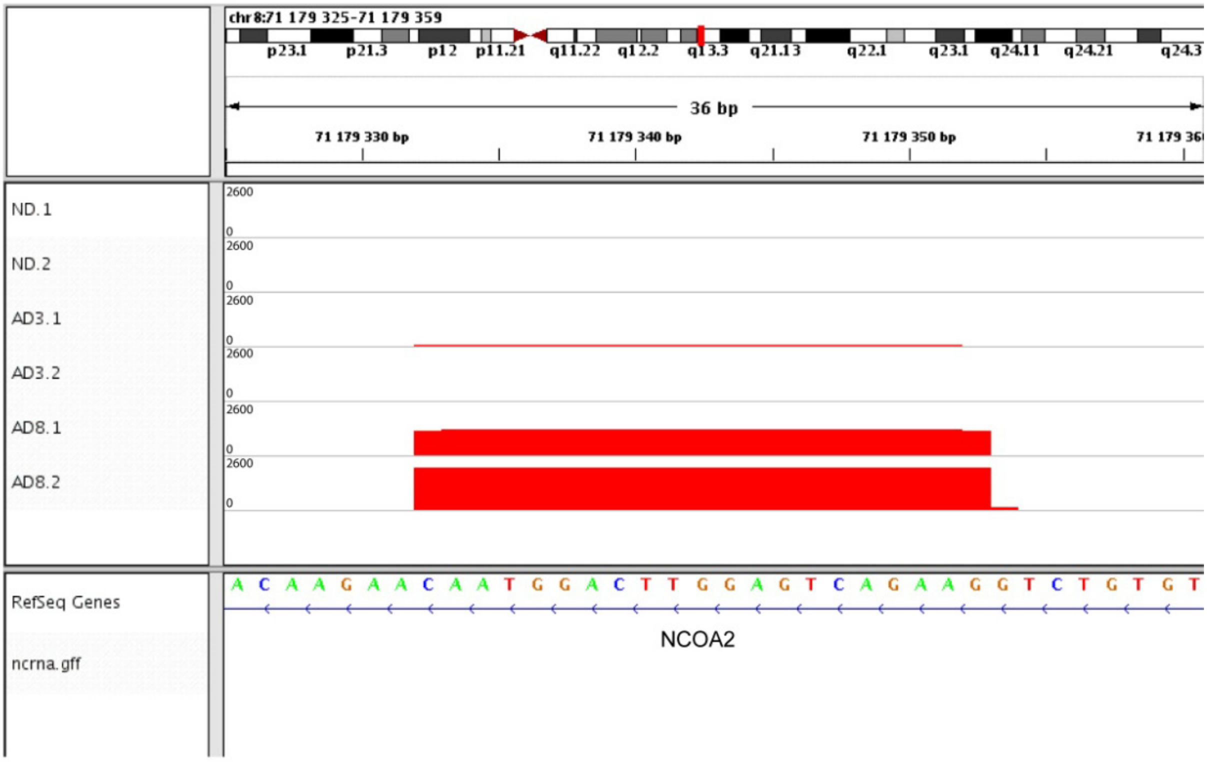

<u>Figure S7:</u> Genome-browser representation of reads matching the 4th intron of NCOA2

For each nucleotide, the corresponding read count was printed. For each panel, the following information is represented, from top to bottom: chromosomal location, genomic coordinates, counts for each sample, transcripts annotations (RefSeq Genes) and non-coding RNA annotations (ncRNA.org). Read counts correspond to undifferentiated (ND.1 and ND.2), day-3 differentiated (AD3.1 and AD3.2) and day-8 differentiated (AD8.1 and AD8.2) hMADS cells samples, with sequencing from 3’ to 5’ end.

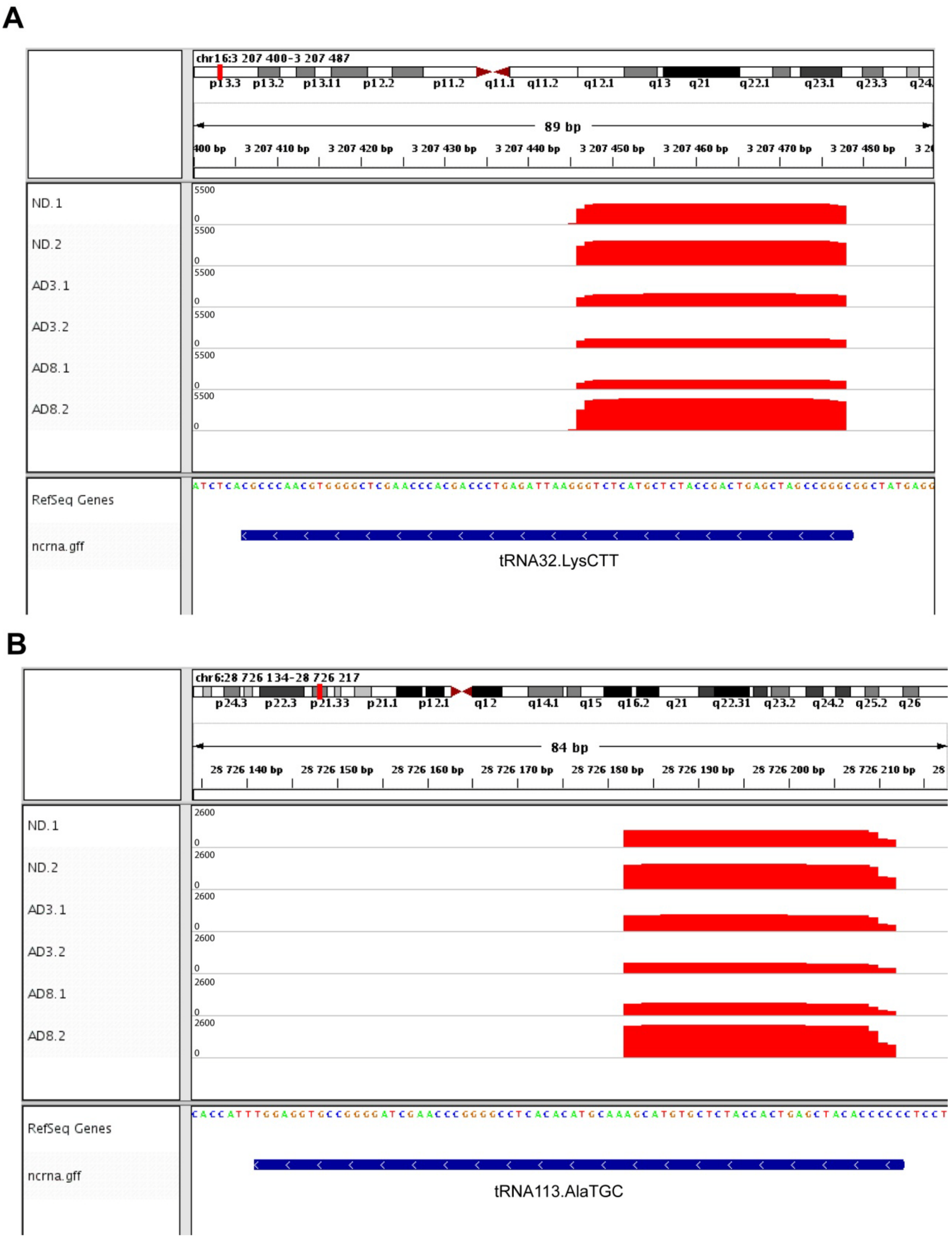

Figure S8: Genome-browser representation of reads matching tRNA32.LysCTT (A) tRNA113.AlaTGC (B)

For each nucleotide, the corresponding read count was printed. For each panel, the following information is represented, from top to bottom: chromosomal location, genomic coordinates,

counts for each sample, transcripts annotations (RefSeq Genes) and non-coding RNA annotations (ncRNA.org). Read counts correspond to undifferentiated (ND.1 and ND.2), day-3 differentiated (AD3.1 and AD3.2) and day-8 differentiated (AD8.1 and AD8.2) hMADS cells samples, with sequencing from 3’ to 5’ end.
