# Supplementary material for: Small RNA sequencing reveals miR-642a-3p as a novel adipocyte-specific microRNA and miR-30 as a key regulator of human adipogenesis
Source: Genome Biol. 2011 Jul 18;12(7):R64. doi: 10.1186/gb-2011-12-7-r64 (PMC3218826; doi:10.1186/gb-2011-12-7-r64)
Supplement: Additional file 11 — Table S5. PCR primer sequences. [file gb-2011-12-7-r64-S11.PDF]

Additional File 11

Table S5: PCR primer sequences

	**Forward primer sequence (given 5' to 3')**	**Reverse primer sequence (given 5' to 3')**
**CEBPB**	AACCAACCGCACATGCAGAT	GGCAGAGGGAGAAGCAGAGAGT
**FABP4**	TGTGCAGAAATGGGATGGAAA	CAACGTCCCTTGGCTTATGCT
**AdipoQ**	GCAGTCTGTGGTTCTGATTCCATAC	GCCCTTGAGTCGTGGTTTCC
**PPARG2**	GAGCCTGCATCTCCACCTTATT	CAGACACGACATTCAATTGCC
**RUNX2**	AGTGGACGAGGCAAGAGTTTCA	GGGTTCCCGAGGTCCATCTA
